# Soleus Muscle Stiffness is Regulated by Scaled Activation to Manage Unpredictable and Predictable Walking Perturbations

**DOI:** 10.1007/s10439-025-03928-3

**Published:** 2025-12-12

**Authors:** Sebastian Bohm, Morteza Ghasemi, Christos Theodorakis, Falk Mersmann, Thomas Roberts, Adamantios Arampatzis

**Affiliations:** 1 Department of Training and Movement Sciences, Humboldt-Universität zu Berlin, Berlin, Germany; 2 Berlin School of Movement Science, Humboldt-Universität zu Berlin, Berlin, Germany; 3 Division of Biology and Medicine, Brown University, Providence, USA

**Keywords:** Muscle–tendon interaction, Unsteady locomotion, Uneven terrain, Motor control, Motor adaptation and learning

## Abstract

During unexpected drop-like gait perturbations, the body’s center of mass (CoM) energy must be absorbed reactively by the leg muscles, challenging muscle–tendon unit (MTU) function and body stability. Anticipation and prior experience may adjust muscle activation in advance to improve the perturbation response. The study's purpose was to investigate the interplay of muscle activation, MTU decoupling mechanisms, and contractile conditions for the CoM energy management during gait challenges.

Kinematics, electromyographic activity (EMG), soleus fascicle length, and total CoM energy were measured during unperturbed walking, unpredictable and adapted (experience-based) drop-like perturbations as well as during hole negotiation. The soleus force–length and force–velocity relationships were also determined to assess the force–length–velocity potential.

CoM energy decreased substantially after touchdown in the hole during both perturbations and hole negotiation, indicating energy absorption by the musculoskeletal system. During the unpredictable perturbation, a rapidly increased EMG activity after drop initiation and an almost isometric fascicle behavior close to optimal length throughout the CoM energy absorption phase was found, despite MTU lengthening. In the adapted perturbation, an initial isometric contraction accompanied by high EMG activity was observed, followed by active fascicle lengthening at decreasing EMG activity. Clear fascicle lengthening in combination with low EMG activity was found during hole negotiation.

These novel findings suggest a regulation of muscle stiffness by scaled activation that tunes the contribution of muscle and tendon to the MTU length changes (i.e., tendon decoupling), to facilitate high fascicle force–length–velocity potentials and tendon energy buffering mechanisms in response to drop-like perturbations and hole negotiation gait.

**Supplementary Information:**

The online version contains supplementary material available at 10.1007/s10439-025-03928-3.

## Introduction

Real-life environments present challenges to locomotor control, including complex terrains, surface irregularities and external perturbations that increase gait instability and the risk of falling [1–3]. To control locomotion and maintain stability, animals utilize feedforward and feedback control mechanisms [4–7]. Visual sensory input allows for long-range anticipatory adjustments in locomotor behavior to prepare for upcoming hazards [8–10]. When encountering an unexpected perturbation, sensory feedback integration reactively modifies ongoing muscle activation patterns through fast short-latency monosynaptic reflexes, longer-latency multi-synaptic responses and later supraspinal neural pathways [7, 11]. Acting in parallel, the intrinsic properties, i.e., force–length and force–velocity relationships, of an activated muscle can provide additional resistance to a perturbation with almost no time delay [12–14]. The experience with a perturbation influences the feedforward motor control through sensorimotor learning processes and leads to adjustments of the muscle activation pattern at the initiation of an expected perturbation [15, 16]. As a result of these predictive adjustments, the consequences of persisting or recurring perturbations are reduced and the feedback control is facilitated [17–19]. Prior experience may also improve the reactive response phase of a similar unexpected perturbation [20, 21].

During an unpredictable drop-like gait perturbation, the body’s center of mass (CoM) energy must be effectively absorbed in the following stance phase to maintain body stability [22, 23]. The distal leg joints are the first to interact with the ground and absorb the major part of kinetic energy when exposed to an unexpected change in surface height [24, 25]. Energy absorption during a drop-like perturbation could require the distal muscles to actively lengthen to dissipate mechanical energy. However, high active fascicle lengthening and long operating length that result from the impact may imply an increased risk for lengthening-induced muscle damage [26–28]. Systematic experiments in turkeys during landing [29, 30] showed that the in-series elastic tendon decouples the muscle fibers from the MTU lengthening (due to joint angle excursion) and stores the body’s energy temporarily as tendon strain energy upon the initial impact. In the subsequent contact phase, the tendon recoils and the muscle fibers dissipate the energy at a lower rate and operate at reduced lengthening velocity, which presumably reduces the risk of muscle damage. Studies in humans suggest a similar protective decoupling behavior in the distal triceps surae muscle–tendon units (MTU) during expected landing tasks [31–33]. The rotation of the muscle fibers within the muscle belly is another potential mechanism of decoupling [34], yet its contribution during human locomotion was shown to be small [35, 36]. Further, MTU decoupling mechanisms (tendon elasticity and fascicle rotation) may also ensure effective operating muscle fascicle length and velocities for force generation required for the CoM energy management; i.e., large fascicle excursions throughout the stance phase can compromise the contractile conditions.

Muscle activation determines the magnitude of decoupling by the elastic tendon because it regulates the muscle stiffness with respect to tendon stiffness. At a low muscle activation level, the muscle stiffness is lower than that of the tendon and MTU excursions at impact following a drop-like perturbation would induce fascicle lengthening. At a higher activation level, the fascicle stiffness exceeds that of the tendon and most of the MTU excursions can be accommodated by tendon elongation [4, 37]. During maximal voluntary isometric contractions, the human Achilles tendon strain is about 5–9% [38, 39], demonstrating that the triceps surae muscle stiffness can be modulated by a scaled activation in this range of strain to induce decoupling by tendon elongation. During a step-down task, Achilles tendon strain estimates of 6% were previously reported [33]. This value is well within the maximal strain range mentioned above. Thus, it can be argued that during drop-like walking perturbations, a scaled muscle activation may effectively regulate the fraction of fascicle lengthening to MTU lengthening by tendon elongation, presumably to prevent high active lengthening and maintain effective operating fascicle conditions during the energy absorption phase. The role of MTU decoupling regulation by muscle activation for the CoM management during drop-like gait perturbations is yet unclear.

Prior knowledge of the perturbation and anticipation will have an effect on the timing and magnitude of muscle activation and thus on muscle stiffness regulation. During expected landings in humans [31, 40–43] and other animals [44–46] distal muscles are anticipatively preactivated prior to ground contact and the activation level remains high in the initial contact phase. The level of preactivation is generally scaled to drop height, stiffening the muscle proportionally to impact intensity prior to touchdown [37, 40, 46]. Further, with experience of a specific gait perturbation, motor learning processes and anticipation adjust the muscle activation pattern to improve the first unpredictable response [16]. Several studies have shown clear proactive and reactive changes in distal muscle EMG activity with repeated exposure to a walking perturbation [47–49], indicating that experience initiates adaptive adjustments in the lower leg muscle activations. The effect of anticipation and experience (learning/adaptation) on the regulation of muscle stiffness by muscle activation for effective MTU decoupling and muscle contractile conditions during drop-like walking perturbations is also unknown at present.

There is currently a limited understanding of how muscle activation, MTU decoupling mechanisms, and muscle contractile conditions interact to manage the CoM energy during the stance phase following unpredictable and predictable drop-like walking perturbations. Therefore, the main objective of the study was to experimentally investigate the CoM energy management at the level of the distal soleus muscle as the main human plantar flexor. For this purpose, we examined whole body kinematics, EMG activity and muscle fascicle length of the soleus, as well as total body energy under four conditions: (1) unperturbed walking; (2) unpredictable (no prior experience) drop-like walking perturbations; (3) adapted (experience-based) drop-like walking perturbations; and (4) hole negotiation gait (anticipatory). Drop-like walking perturbations were applied, as walking is the most common fall-related activity in humans (~47% [50]) and misplaced steps such as drop-like perturbations can account for 12 to 22% of all fall cases [51, 52]*.* We further determined the force–length and force–velocity potential of the soleus during the investigated tasks by mapping the operating length and velocities on the experimentally assessed force–length and force–velocity curves. We hypothesized that fast neuromuscular responses following the unpredictable drop-like perturbation would regulate muscle stiffness and limit muscle fascicle lengthening during the first part of the stance phase. We further expected proactive adjustments in muscle EMG activity patterns during repeated perturbations and hole negotiation gait, which would influence the MTU decoupling mechanisms and muscle fascicle behavior, enabling a more effective management of the CoM energy. Information on reactive and experience-based/anticipatory adjustments in neuromuscular control is important for interventions on fall and injury prevention as well as the design of exoskeletons, legged robots, and assistive devices.

## Materials and Methods

### Experimental Design

Nineteen young and healthy adults (10 females, 9 males) participated in the present study (body height 171 ± 10 cm, body mass 68 ± 11 kg, age 24 ± 4 years, mean ± standard deviation). Any history of recent systemic, neuromuscular or skeletal impairments and injuries was defined as exclusion criteria. The ethics committee of the Humboldt-Universität zu Berlin approved the study (HU-KSBF-EK_2022_0032) and the participants gave written informed consent in accordance with the Declaration of Helsinki.

**Fig. 1:**
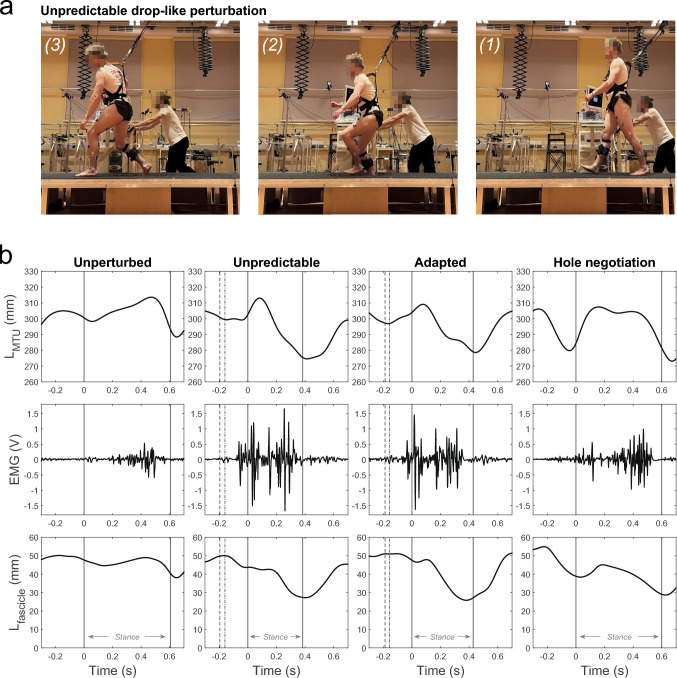
**a)** Illustration of the experimental setup. An electronic perturbation device with a hidden drop-plate (15 cm depth) was used to introduce unpredictable (example image series on top) and adapted drop-like perturbation during walking on a gangway. **b)** Representative experimental data from one exemplarily participant of the soleus muscle–tendon unit (MTU) length, electromyographic activity (EMG), and ultrasound-based muscle fascicle length during unperturbed level walking (column left), the unpredictable drop-like walking perturbation (mid-left column), the adapted drop-like walking perturbation (mid-right column), and the hole negotiation (right column). During perturbations (mid panels), the first dotted line indicates the touchdown on the plate and the second dashed line the drop initiation of the plate. The solid vertical lines indicate the right touchdown, i.e., after the plate drop in the hole during perturbations, and the consecutive toe off

Participants walked barefoot on an 18 m long, 1 m broad, and 0.4 m high customized gangway at preferred speed (1.22 ± 0.11 m/s*,* fig. 1). The gangway integrated a hidden electronic perturbation device in the second half with a releasable floor plate (70x46 cm), which was placed on a force plate (sampling frequency 1 kHz, AMTI BP600, Watertown, MA, USA). The drop height of the plate was set to 15 cm to induce a significant drop-like perturbation during walking (fig. 1). The starting position of the participants was adjusted during familiarization to ensure that participants always stepped onto the plate with their right foot. The perturbation was introduced by releasing the plate during the initial stance phase of the right leg (i.e., ~ 30 ms after touchdown), triggered based on a spatial threshold in the walking direction upon passing by the calcaneus marker. The participants were secured by a safety harness, connected with a moveable carriage at the ceiling (fig. 1).

The experimental trial protocol included a familiarization phase (15 to 20 walking trials), three unperturbed baseline walking trials, an unpredictable drop-like walking perturbation without prior experience, five further predictable drop-like walking perturbations (thus, with prior experience), and finally five trials where the participants walked through the visible hole without being perturbed. Although the participants were informed about the occurrence of a gait perturbation at a certain time point of the experiment, the first perturbation was unpredictable in terms of its spatial and temporal characteristics, without prior experience and without knowing on which trial it would be applied. In the following repeated perturbation trials the location of the plate was marked and the participants received the information that the plate will be released upon touchdown of their right foot; thus, the perturbation was predictable and the participants adapted their gait based on the repeated experience [17]. The first unperturbed baseline walking trial, the unpredictable perturbation trial (i.e., no prior experience), the last predictable perturbation trial (i.e., adapted based on prior experience), and the fifth walk-through-the-hole trial were used for further analysis. After completing the gait protocol, the soleus force–fascicle length relationship was determined experimentally in a separate measurement. All measured and calculated parameters are summarized and defined in supplementary Table1.

## Kinematics and Total Body Energy

During walking, whole body kinematics were captured by a motion capture system integrating 19 cameras (Vicon Motion Systems, Oxford, UK; 250 Hz, 0.017 mm trueness) and 3D positions of 21 anatomically referenced reflective markers (two at the front and back of the cranial bone, 7^th^ cervical vertebra, left and right acromion, joint line of the elbow and wrist, greater trochanter, lateral femoral epicondyle and malleolus, second metatarsal, and tuber calcanei). The 3D marker coordinates were processed by a 2^nd^-order Butterworth low-pass filter with a 6 Hz cut-off frequency. For the ankle angle, 0° indicates the shank perpendicular to the foot and values < 0° indicate dorsiflexion. For the knee joint, an angle of 180° indicates full knee extension, with joint angles of less than 180° indicating flexion. The touchdown and toe off of the perturbed right leg were detected using the vertical ground reaction force from the force plate (threshold of 40 N with respect to the unloaded force plate). The touchdown in the hole after the plate release during the perturbation trials was determined by the vertical acceleration of the metatarsal marker [53] because of plate drop artifacts on the ground reaction force.

The total body energy of the individuals’ center of mass (CoM) was calculated as (equation 1):

1 $$E_{CoM} = mgH_{CoM} + \frac{1}{2}mV_{CoM}^{2}$$

where m is the body mass, g is the acceleration of gravity, H_CoM_ is the height of the CoM, and V_CoM_ is the CoM velocity. The masses of the segments and their respective positions in order to calculate the CoM were taken from Dempster [54]. It should be noted that we did not consider the rotational energy of each segment or its kinetic energy relative to the CoM in our total CoM energy. Due to the low moment of inertia and angular velocity of the segments, as well as their relative velocity with respect to the CoM, the contribution of these energy sources is quite small, resulting in minimal differences in the total CoM energy [55, 56].

## Soleus Muscle Fascicle Behavior and Electromyographic Activity

An ultrasound device (Aloka Prosound Alpha 7, Tokyo, Japan) was mounted on a wagon and manually pushed alongside the participants during walking. The soleus muscle fascicles were captured at 146 Hz by a 6 cm linear array ultrasound probe (UST-5713 T, 13.3 MHz) synchronously with the kinematic data. The probe was placed on the medial aspect of the soleus muscle belly using a custom neoprene–plastic cast. The fascicle length was determined from the ultrasound images as the average of multiple fascicle portions automatically identified over the entire field of view between the two semi-automatically tracked upper and deeper aponeuroses [57, 58]. Each image was visually controlled and manual corrections were made if necessary. The raw fascicle length was processed using a second-order low-pass Butterworth filter with a 6 Hz cut-off frequency. The pennation angle was defined by the angle between the deeper aponeurosis and the average fascicle. The soleus muscle–tendon unit (MTU) length changes were calculated by multiplying the individual instantaneous Achilles tendon lever arm (see below) and the ankle joint angle changes [59]. The initial MTU length was determined by a regression equation [60] at a 0° ankle joint angle. The belly length changes of the soleus muscle were calculated as the difference of consecutive products of fascicle length and the cosine of the pennation angle [61]. Note that this does not reflect the length of the entire soleus muscle belly but indicates the projection of the instant fascicle length onto the plane of the MTU [62]. The velocity of the muscle fascicles, belly, and MTU was calculated as the first derivative of length over time (shortening denoted with a negative sign).

Surface EMG of the soleus muscle was measured at 1000 Hz using a wireless electromyography system (Myon m320RX, Myon AG, Baar, Switzerland). The electrodes were placed after careful skin preparation on the medial portion of the soleus according to current guidelines (www.seniam.org). The EMG data were processed using a fourth-order high-pass Butterworth filter with a 20 Hz cut-off frequency, a full-wave rectification and then a low-pass filter with a 20 Hz cut-off frequency. The EMG activity was normalized to the individual maximum EMG activity obtained during a maximum isometric plantar flexion contraction at a 0° ankle joint angle. A representative dataset of one participant is illustrated in figure 1.

## Assessment of the Soleus Muscle Intrinsic Properties

**Fig 2:**
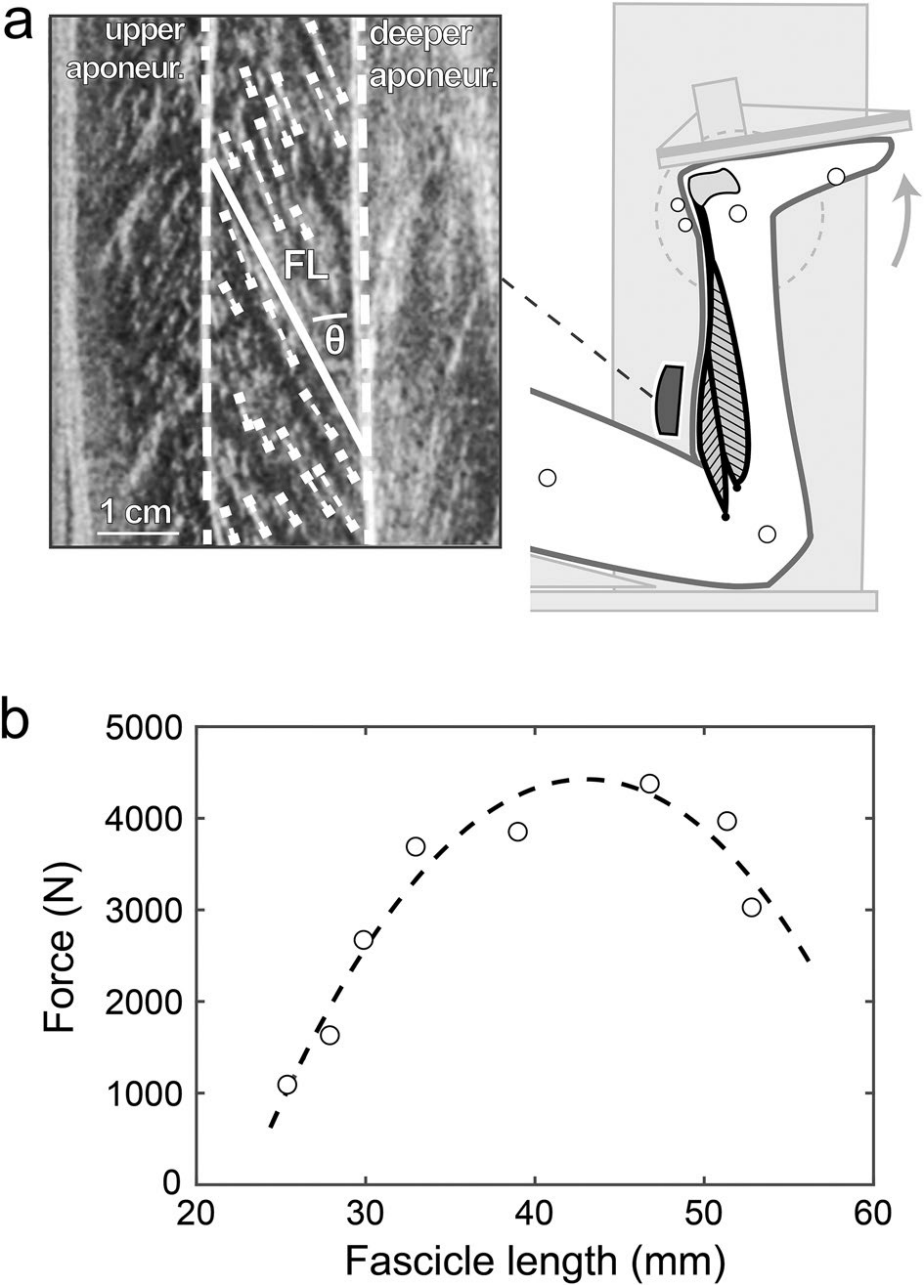
Experimental setup for the determination of the soleus force–fascicle length relationship. **a)** Maximum isometric plantar flexions (MVC) at eight different joint angles were performed on a dynamometer. During the MVCs, ultrasound images of the soleus were recorded and a representative muscle fascicle length (FL, solid line) was calculated based on multiple fascicle portions (short dashed lines) and the pennation angle (θ) was defined as the angle between F and the deeper aponeurosis. **b)** An individual soleus force–fascicle length relationship was derived from the MVCs (circles) by means of a second-order polynomial fit (dashed line, MVCs, and curve of one representative participant)

The force–length relationship of the soleus muscle was determined experimentally by a combination of dynamometry and ultrasound in a second part of the experiment immediately after the walking perturbations (fig. 2). The ultrasound probe, EMG electrodes and required markers remained attached between both measurement parts (walking perturbations and muscle assessment). The participants lay prone on the bench of a dynamometer (Biodex Medical, Syst. 3, Inc., Shirley, NY, USA) with the knee joint flexed at ∼60° to restrict the contribution of the bi-articular m. gastrocnemii to the plantar flexion moment (fig. 2) [63, 64]. Following a standardized warm-up, participants performed eight maximum voluntary fixed-end isometric plantar flexion contractions (MVCs) at different ankle joint angles [65]. The ankle joint angles were equally distributed from 15 deg plantar flexion to the individual maximal dorsiflexion angle and applied in random order. The resultant ankle joint moments were determined by means of inverse dynamics, considering the effects of gravitational moments and misalignments between the joint axis and dynamometer axis [66]. The required kinematic data were recorded by means of a Vicon motion capture system integrating 11 cameras at 250 Hz. Furthermore, passive moments and the contribution of the antagonistic muscles during the plantar flexion contractions were considered. To assess the contribution of antagonistic muscles, we used the EMG-based approach by Mademli et al. [67], taking into account the force–length dependency of the antagonists [65]. The force applied to the Achilles tendon during the MVCs was calculated from the plantar flexion moment and the instantaneous Achilles tendon lever arm. The lever arm was defined as the shortest perpendicular distance between the rotational ankle joint axis, given by the two malleoli markers, and the tendon’s line of action. The tendon’s line of action was determined by the vector between the calcaneal marker and a second marker placed 2 cm proximal to the calcaneus over the free Achilles tendon (fig. 2). The marker radius (both Ø 6 mm) and the distance from the skin surface to the midline of the tendon were considered, respectively. The midline of the tendon was defined as the center of the outer anterior and posterior tissue layer of the tendon, measured in a longitudinal ultrasound image.

The fascicle length of the soleus muscle was synchronously captured by means of ultrasonography and the fascicle length was measured during the MVC as described above. A second-order polynomial fit of the quantified forces and fascicle lengths at the plateau of the MVCs at the different ankle joint angles was used to determine an individual force–fascicle length relationship of the soleus muscle (fig. 2). The maximum muscle force applied to the tendon (F_max_) and optimal fascicle length for force generation (L_0_) were derived from the polynomial function accordingly [65, 68]. The force–velocity relationship of the soleus was assessed using the dimensionless form [69] of the Hill equation [70] and the required maximal shortening velocity (V_max_) and the constant a_rel_ were taken from the literature. The V_max_ was taken from Luden et al., reporting values for single soleus type 1 fibers of 0.77 L_0_/s and for type 2 fibers of 2.91 L_0_/s measured at 15 °C [71]. The values were then corrected for physiological temperature conditions (37 °C) using reported coefficients [72], predicting V_max_ as 4.4 L_0_/s for type 1 and 16.8 L_0_/s for type 2 fibers. Finally, an average fiber type distribution (type 1: 81.3%, type 2: 18.7%) of the human soleus muscle reported in the literature was considered [71, 73–75] to calculate V_max_ as 6.77 L_0_/s. The experimentally determined individual L_0_ was used to calculate an individual estimate of V_max_. The constant a_rel_ was calculated by 0.1 + 0.4 type 2 fiber percentage as 0.175 [76]. Using the force–length and force–velocity relationships, we quantified the individual force–length potential (fraction of the soleus maximum force according to the force–length relationship) and force–velocity potential (fraction of the soleus maximum force according to the force–velocity relationship) as a function of the fascicle operating length and velocity during the unperturbed, perturbed, and hole negotiation walking trials [65]. The force–length–velocity potential was then calculated by the product of the force–length and force–velocity potential.

## Assessment of Decoupling within the Soleus Muscle–Tendon Unit

The decoupling of the soleus muscle fascicles from the MTU was quantified using the decoupling coefficients formulated by Bohm et al. [35] separately for the tendon elasticity decoupling (DC_tendon_, equation 2), fascicle rotation decoupling (DC_belly_, equation 3), and for the overall decoupling of MTU and fascicle that includes both components (DC_MTU_, equation 4) as:

2 $$DC_{tendon} \left( t \right) = {\raise0.7ex\hbox{${\left| {V_{MTU} \left( t \right) - V_{belly} \left( t \right)} \right|}$} \!\mathord{\left/ {\vphantom {{\left| {V_{MTU} \left( t \right) - V_{belly} \left( t \right)} \right|} {V_{\max } }}}\right.\kern-0pt} \!\lower0.7ex\hbox{${V_{\max } }$}}$$

3 $$DC_{belly} \left( t \right) = {\raise0.7ex\hbox{${\left| {V_{belly} \left( t \right) - V_{fascicle} \left( t \right)} \right|}$} \!\mathord{\left/ {\vphantom {{\left| {V_{belly} \left( t \right) - V_{fascicle} \left( t \right)} \right|} {V_{\max } }}}\right.\kern-0pt} \!\lower0.7ex\hbox{${V_{\max } }$}}$$

4 $$DC_{MTU} \left( t \right) = {\raise0.7ex\hbox{${\left| {V_{MTU} \left( t \right) - V_{fasicle} \left( t \right)} \right|}$} \!\mathord{\left/ {\vphantom {{\left| {V_{MTU} \left( t \right) - V_{fasicle} \left( t \right)} \right|} {V_{\max } }}}\right.\kern-0pt} \!\lower0.7ex\hbox{${V_{\max } }$}}$$

Here *V(t)* is the MTU, belly, and fascicle velocity during the stance phase. These decoupling coefficients were used to investigate the time-varying decoupling process between MTU and belly, belly and fascicles, and MTU and fascicles throughout the stance phase of unperturbed and perturbed walking, respectively [35].

## Statistics

A linear mixed model with participants treated as a random effect and task type as a fixed effect (unperturbed walking, unpredictable perturbation, adapted perturbation, hole negotiation) was used separately for the phase of MTU lengthening and MTU shortening or the entire stance phase. In case of a main effect of trial type, a Benjamini–Hochberg corrected post hoc analysis was conducted and adjusted p-values are reported. In some parameters, the normal distribution of the normalized residuals was not given, as tested by the Shapiro–Wilk test, yet linear mixed models are robust against violations of the normality assumption (Jacqmin-Gadda et al., 2007). For the comparison of the time from touchdown on the plate to the initiation of the plate drop and the time from plate drop initiation to the touchdown after the drop of the plate during the unpredictable and adapted perturbation a t-test for dependent samples was used. The level of significance was set to α = 0.05 and the statistical analyses were performed using R (RStudio, v.2022.07.1, PBC, Boston, MA, United States) and the *nlme* and *emmeans* packages. Furthermore, SPM (one sample t-test, α = 0.05) was used to test for differences of the fascicle and MTU velocity to zero throughout the stance phase of the different trials. The SPM analysis was conducted using the software package spm1D (version 0.4, http://www.spm1d.org, [77]). Effect sizes were calculated as Cohen’s d using the R function eff_size of the emmeans package. Effect sizes of 0.2 ≤ d < 0.5 are referred to as small, 0.5 ≤ d < 0.8 as medium, and d ≥ 0.8 as large [78].

## Results

The experimentally determined L_0_ of the soleus was 45.3 ± 7.0 mm, the corresponding F_max_ was 3,772 ± 829 N and V_max_ was 307 ± 48 mm/s. The stance time (ground contact) was significantly reduced during the unpredictable (432 ± 114 ms) and adapted perturbation (520 ± 68 ms) compared with unperturbed walking (665 ± 47 ms, d = ) and hole negotiation (647 ± 65 ms), yet shortest during the unpredictable perturbation (all p < 0.001). The time from touchdown on the plate to the initiation of the plate drop was 38.7 ± 14.1 ms in the unpredictable and 29.3 ± 8.9 ms in the adapted perturbation (p = 0.0168) and the time from plate drop initiation to the touchdown after the plate drop in the hole was 154.7 ± 11.5 ms and 144.2 ± 8.5 ms (p < 0.001).

**Fig. 3:**
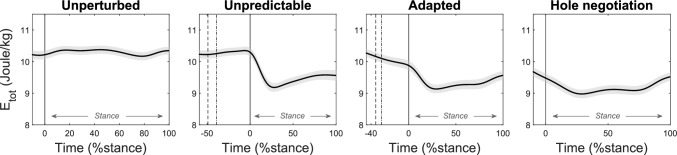
Total energy (E_tot_) of the participants’ center of mass during unperturbed level walking (left), the unpredictable drop-like walking perturbation (mid-left), the adapted walking perturbation (mid-right), and the hole negotiation (right). During perturbations (mid panels), the first dotted line indicates the touchdown on the plate and the second dashed line the drop initiation of the plate. The solid vertical line indicates the right touchdown, i.e., after the plate drop in the hole during perturbations. The stance phase was defined from touchdown until toe off (0–100 %stance, *n* = 19, mean ± standard error)

In unperturbed walking, the peak-to-peak range of the total CoM energy was small throughout the stance phase, but it increased during both perturbation tasks and hole negotiation gait (fig. 3). During the unpredictable and adapted perturbation as well as during hole negotiation, the total CoM energy decreased in the first part of the stance phase, indicating energy absorption by the musculoskeletal system (fig. 3). The decrease was greater during the unpredictable (1.36 ± 0.5 J/kg) compared with the adapted perturbation (0.82 ± 0.27 J/kg, p < 0.001) and smallest during hole negotiation (0.52 ± 014 J/kg, both p < 0.003). In the second part of stance, the total CoM energy increased in the unpredictable (0.62 ± 0.45 J/kg) and adapted perturbation (0.52 ± 0.20 J/kg) as well as hole negotiation (0.57 ± 0.15 J/kg), demonstrating energy production by the musculoskeletal system, with no significant differences between these tasks (all p > 0.200, fig. 3).

**Fig. 4:**
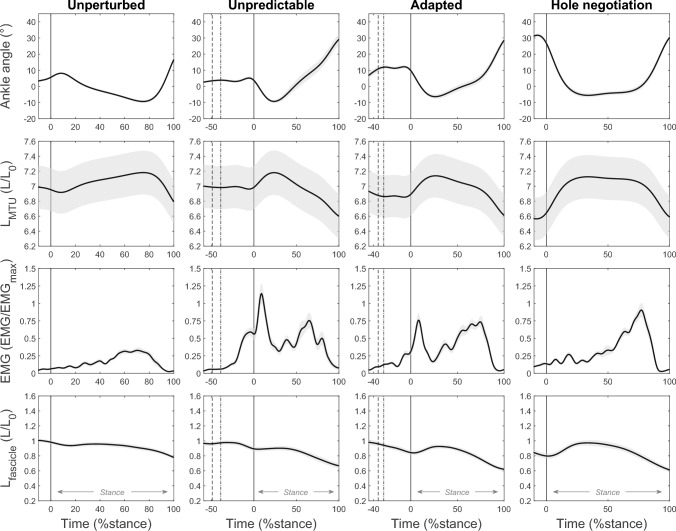
Ankle joint angle, soleus muscle–tendon unit length (L_MTU_), electromyographic activity (EMG), and fascicle length (L_fascicle_) during the unperturbed level walking (column left), the unpredictable drop-like walking perturbation (mid-left column), the adapted walking perturbation (mid-right column), and the hole negotiation (right column). L_MTU_ and L_fascicle_ are normalized to optimal fascicle length (L_0_) and EMG activity is normalized to a maximum voluntary isometric contraction (EMG_max_). During perturbations (mid panels), the first dotted line indicates the touchdown on the plate and the second dashed line indicates the drop initiation of the plate. The solid vertical line indicates the right touchdown, i.e., after the plate drop in the hole during perturbations. The stance phase was defined from touchdown until toe off (0–100 %stance, *n* = 19, mean ± standard error)

The ankle joint dorsiflexed directly after touchdown in the hole in the unpredictable and adapted perturbation as well as during hole negotiation (fig. 4). Accordingly, the soleus MTU lengthened following touchdown in the hole during all three tasks (fig. 4). The MTU lengthening magnitude was smaller during the unpredictable compared with the adapted perturbation (p = 0.045) and greatest during hole negotiation (both p < 0.001), where also the lengthening duration was longer (p < 0.001, p = 0.007, fig. 4, table 1). At touchdown in the hole, the MTU length was shorter during the adapted compared with the unpredictable perturbation (6.98 ± 1.25 L/L_0_, 6.85 ± 1.22 L/L_0_, p < 0.001) and shortest during hole negotiation (6.64 ± 1.23 L/L_0_, both p < 0.001, fig. 4), due to increased plantar flexion (fig. 4). An increased plantar flexion was found already at touchdown in the level before the plate drop in the adapted perturbation (12.5 ± 5.3°) and hole negotiation (26.8 ± 4.5°) compared with the unpredictable perturbation (4.0 ± 2.5°, p < 0.001, fig. 4). In the second part of the stance phase, the MTU shortened continuously until toe off during the perturbations and hole negotiation, with no differences between tasks (all p > 0.100). During unperturbed walking, plantar flexion and MTU shortening occurred comparably later during stance (all p > 0.100) and were on average less (all p > 0.100, fig. 4, table 1).

**Table 1:** Duration, muscle–tendon unit (MTU) length changes (∆L), mean MTU velocity (V), mean electromyographic activity (EMG), muscle fascicle length changes, mean fascicle velocity, mean decoupling coefficients for tendon compliance (DC_tendon_), fascicle rotation (DC_belly_), and the entire MTU (DC_MTU_) as well as mean force–length (λ_L_), mean force–velocity potential (λ_V_), and mean force–length–velocity potential (λ_LV_) for the phase of MTU lengthening and shortening during the unperturbed level walking, the unpredictable drop-like walking perturbation, the adapted drop-like walking perturbation, and the hole negotiation, respectively (n = 19, mean ± standard deviation). MTU and fascicle length are normalized to optimal fascicle length (L_0_) and velocities are normalized to the fascicles' maximum shortening velocity (V_max_). EMG activity is normalized to a maximum voluntary isometric contraction (EMG_max_)

	***MTU lengthening***				***MTU shortening***			
	**Unperturbed**	**Unpredictable**	**Adapted**	**Hole negotiation**	**Unperturbed**	**Unpredictable**	**Adapted**	**Hole negotiation**
Duration (ms) *	496 ± 44^a^	148 ± 158^b^	243 ± 217^b^	425 ± 290^a^	169 ± 22^a^	284 ± 183^a^	276 ± 210^a^	222 ± 291^a^
∆ L_MTU_ (L/L_0_) * ^#^	0.230 ± 0.068^a,b^	0.226 ± 0.085^a^	0.274 ± 0.081^b^	0.500 ± 0.083^c^	0.391 ± 0.078^a^	0.611 ± 0.175^b^	0.556 ± 0.127^b^	0.554 ± 0.087^b^
V_MTU_ (V/V_max_) * ^#^	0.067 ± 0.018^a^	0.294 ± 0.083^b^	0.252 ± 0.103^b^	0.292 ± 0.117^b^	-0.339 ± 0.084^a^	-0.305 ± 0.094^a,b^	-0.263 ± 0.086^b,c^	-0.246 ± 0.069^c^
EMG (EMG/EMG_max_) * ^#^	0.18 ± 0.07^a^	0.73 ± 0.30^b^	0.42 ± 0.20^c^	0.21 ± 0.07^a^	0.14 ± 0.08^a^	0.41 ± 0.14^b^	0.45 ± 0.14^b^	0.44 ± 0.17^b^
∆ L_fascicle_ (L/L_0_) * ^#^	-0.080 ± 0.104^a^	0.016 ± 0.068^b^	0.087 ± 0.099^c^	0.150 ± 0.098^d^	0.124 ± 0.073^a^	0.241 ± 0.086^b^	0.308 ± 0.111^c^	0.336 ± 0.090^c^
V_fascicle_ (V/V_max_) * ^#^	-0.025 ± 0.031^a^	0.009 ± 0.085^a^	0.078 ± 0.097^b^	0.081 ± 0.064^b^	-0.108 ± 0.067^a^	-0.121 ± 0.049^a^	-0.143 ± 0.056^a^	-0.147 ± 0.048^a^
DC_tendon_ * ^#^	0.11 ± 0.03^a^	0.28 ± 0.10^b^	0.21 ± 0.07^c^	0.24 ± 0.12^b,c^	0.25 ± 0.08^a^	0.19 ± 0.07^b^	0.17 ± 0.06^b^	0.16 ± 0.04^b^
DC_belly_ * ^#^	0.005 ± 0.003^a^	0.010 ± 0.008^a,b^	0.013 ± 0.010^b,c^	0.019 ± 0.019^c^	0.008 ± 0.007^a^	0.017 ± 0.011^b^	0.019 ± 0.013^b^	0.020 ± 0.017^b^
DC_MTU_ * ^#^	0.11 ± 0.03^a^	0.29 ± 0.10^b^	0.21 ± 0.07^c^	0.24 ± 0.11^b,c^	0.25 ± 0.08^a^	0.20 ± 0.07^b^	0.17 ± 0.06^b^	0.17 ± 0.04^b^
λ_L_ * ^#^	0.96 ± 0.04^a^	0.93 ± 0.06^a,b^	0.91 ± 0.09^b^	0.90 ± 0.09^b^	0.91 ± 0.07^a^	0.82 ± 0.14^b^	0.81 ± 0.12^b^	0.82 ± 0.11^b^
λ_V_ *	0.88 ± 0.12^a^	0.96 ± 0.23^a,b^	1.07 ± 0.14^b^	1.06 ± 0.13^b^	0.61 ± 0.18^a^	0.60 ± 0.13^a^	0.54 ± 0.14^a^	0.52 ± 0.11^a^
λ_LV_^#^	0.84 ± 0.12^a^	0.89 ± 0.22^a^	0.98 ± 0.16^a^	0.95 ± 0.12^a^	0.56 ± 0.16^a^	0.50 ± 0.15^a,b^	0.44 ± 0.11^b^	0.43 ± 0.10^b^

^*^ Significant main effect of trial during MTU lengthening (p < 0.05)

^#^ Significant main effect of trial during MTU shortening (p < 0.05)

Post hoc analysis: Different letters denote significant differences between walking trials (p < 0.05)

In the unpredictable perturbation, the EMG activity increased after the plate drop initiation, whereas during the adapted perturbation, it increased before the plate drop initiation (fig. 4). The average EMG activity between touchdown on the plate in the level and the shortly later plate drop initiation was slightly but significantly higher in the adapted (0.123 ± 0.069 EMG/EMG_max_) compared with the unpredictable perturbation (0.075 ± 0.062 EMG/EMG_max_, p = 0.010). Yet, the EMG activity at subsequent touchdown in the hole was significantly higher in the unpredictable (0.560 ± 0.344 EMG/EMG_max_) compared with the adapted perturbation (0.314 ± 0.193 EMG/EMG_max_, p < 0.001). Similarly, the average EMG activity during MTU lengthening was greater during the unpredictable compared with the adapted perturbation (p < 0.001, table 1). In unperturbed walking and hole negotiation, the EMG activity was significantly lower at touchdown and throughout the entire MTU lengthening phase compared with the unpredictable and adapted perturbation (all p < 0.001, fig. 4, table 1). The maximum EMG activity in these two tasks occurred during the MTU shortening phase (i.e., push-off), where also a second peak in the EMG activity was found in the unpredictable and adapted perturbation (fig. 4). The average EMG activity during the MTU shortening phase was not different between hole negotiation, unpredictable, and adapted perturbation (all p > 0.400), but was lowest during unperturbed walking (all p < 0.001; fig. 4, table 1).

**Fig. 5:**
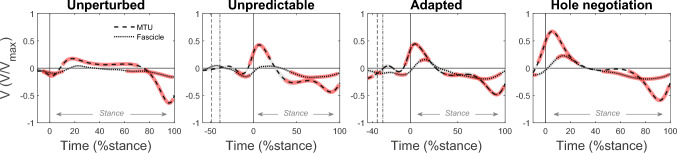
Soleus muscle–tendon unit (MTU) and fascicle velocity (V, positive sign indicates lengthening) during the stance phase of unperturbed level walking (left), the unpredictable drop-like walking perturbation (mid-left), the adapted walking perturbation (mid-right), and the hole negotiation (right). V is normalized to the fascicles’ maximum shortening velocity (V_max_). During perturbations (mid panels), the first dotted line indicates the touchdown on the plate and the second dashed line indicates the drop initiation of the plate. The solid vertical line indicates the right touchdown, i.e., after the plate drop in the hole during perturbations. The stance phase was defined from touchdown until toe off (0–100% stance). Intervals with a significant fascicle and MTU velocity difference to zero are illustrated by the red underlay (SPM{t}, p < 0.05, *n* = 19, mean ± standard error)

**Fig. 6:**
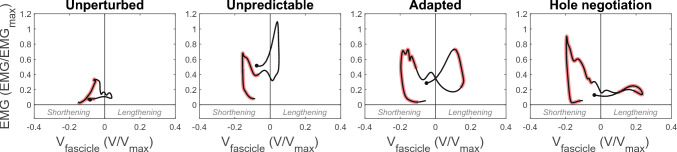
Soleus muscle electromyographic activity (EMG) and fascicle velocity (V, positive sign indicates lengthening) during the stance phase of unperturbed level walking (left), the unpredictable drop-like walking perturbation (mid-left), the adapted walking perturbation (mid-right), and the hole negotiation (right). Circles indicate the beginning of the line at touchdown and thus the operating direction during stance. Intervals during the stance phase with a significant fascicle velocity difference to zero are indicated by the red underlay (SPM{t}, p < 0.05, *n* = 19)

Despite the lengthening of the MTU after touchdown in the hole during the unpredictable and adapted perturbation, the fascicles operated with only small length changes and quite close to L_0_ in this phase. Fascicle lengthening was slightly greater during the adapted perturbation (p = 0.027, fig. 4, table 1). During hole negotiation, the lengthening of the fascicles during the MTU lengthening phase was greater compared with the unpredictable and adapted perturbation (p < 0.001, p = 0.042, fig. 4, table 1). The fascicle velocity during the MTU lengthening phase was not significantly different from zero during the unpredictable perturbation (p > 0.05, fig. 5), while there was a short interval of significant differences to zero (i.e., 9–21 %stance) in the adapted perturbation and a larger interval in the hole negotiation task (i.e., 8–24 %stance, fig. 5). The non-significant difference from zero of the fascicle velocity in the unpredictable perturbation was accompanied by a high EMG activity of the soleus muscle (fig. 6). The significant differences from zero in fascicle lengthening velocity during the adapted perturbation coincide with the decrease in EMG activity (fig. 6). During hole negotiation, the EMG activity was low throughout the entire MTU lengthening phase and the soleus fascicle lengthening velocity was higher than during the two perturbations (fig. 6). Changes in fascicle length during MTU lengthening in unperturbed walking were smaller in comparison with the other three tasks (all p < 0.004; fig. 4; table 1) and fascicle velocity was mostly not significantly different from zero (p > 0.05; fig. 5). During the MTU shortening phase, the soleus fascicles shortened continuously in all investigated locomotor tasks, with the smallest shortening in unperturbed walking (all p < 0.001). Fascicle shortening was less during the unpredictable perturbation compared with the adapted perturbation and hole negotiation (p = 0.012, p < 0.001; fig. 4, table 1). In all four tasks, the fascicle shortening velocity was significantly different from zero for most of the MTU shortening phase (p < 0.05, fig. 5). The average fascicle shortening velocity in this phase was not significantly different between tasks (all p > 0.086, table 1).

**Fig. 7:**
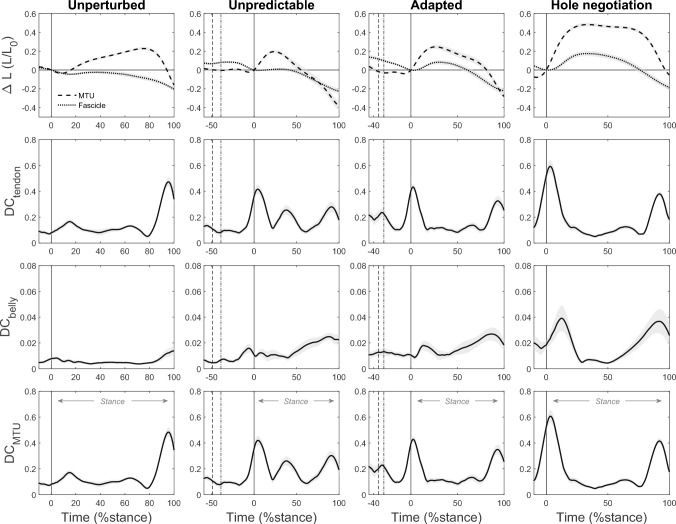
Soleus muscle–tendon unit (MTU) and fascicle length changes (∆L) with respect to the length at touchdown (0 %stance) and corresponding velocity decoupling coefficients for tendon compliance (DC_tendon_) and fascicle rotation (DC_belly_) and the entire MTU (DC_MTU_) during unperturbed level walking (column left), the unpredictable drop-like walking perturbation (mid-left column), the adapted walking perturbation (mid-right column), and the hole negotiation (right column). During perturbations (mid panels), the first dotted line indicates the touchdown on the plate and the second dashed line indicates the drop initiation of the plate. The solid vertical line indicates the right touchdown, i.e., after the plate drop in the hole during perturbations. The stance phase was defined from touchdown until toe off (0–100 %stance, *n* = 19, mean ± standard error)

In all four tasks, there was a clear decoupling of the fascicle length changes from the MTU, mainly by decoupling due to tendon elasticity (fig. 7, table. 1). During MTU lengthening, DC_MTU_ and DC_tendon_ were greater in the unpredictable perturbation compared with the adapted perturbation (both p = 0.006), yet both perturbation tasks did not differ significantly from hole negotiation (all p > 0.082). During unperturbed walking, the two decoupling coefficients showed the lowest values (all p < 0.001, fig. 7, table. 1). DC_belly_ was lower during unperturbed walking compared with the adapted perturbation (p = 0.018) and hole negotiation (p < 0.001) as well as lower during the unpredictable perturbation compared with hole negotiation (p = 0.009, fig. 7, table. 1). During the MTU shortening phase, DC_tendon_, DC_belly,_ and DC_MTU_ did not differ significantly between the unpredictable perturbation, adapted perturbation, and hole negotiation (all p > 0.100). However, DC_MTU_ and DC_tendon_ were significantly greater and DC_belly_ significantly lower in unperturbed walking compared with the other tasks (all p < 0.005; fig. 7, table. 1).

**Fig. 8:**
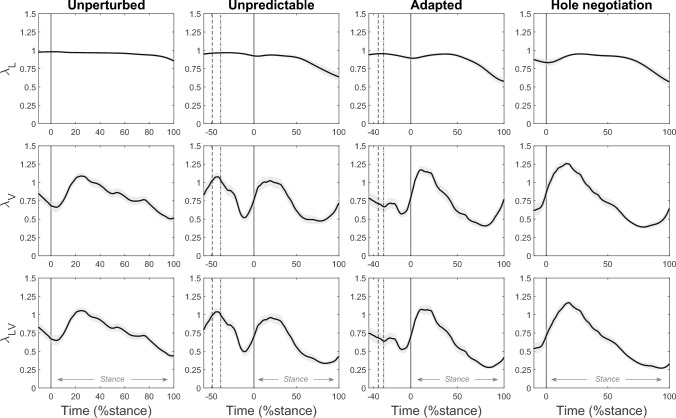
Soleus muscle force–length potential (λ_L_), force–velocity potential (λ_V_), and force–length–velocity potential (λ_LV_) during unperturbed level walking (column left), the unpredictable drop-like walking perturbation (mid-left column), the adapted walking perturbation (mid-right column), and the hole negotiation (right column). During perturbations (mid panels), the first dotted line indicates the touchdown on the plate and the second dashed line indicates the drop initiation of the plate. The solid vertical line indicates the right touchdown, i.e., after the plate drop in the hole during perturbations. The stance phase was defined from touchdown until toe off (0–100 %stance, *n* = 19, mean ± standard error)

The force–length, force–velocity, and force–length–velocity potentials of the soleus fascicles were high (0.84–1.07) during the MTU lengthening phase in all tasks (fig. 8, table 1). There were no significant differences between the two perturbation and the hole negotiation tasks for the three potentials (all p > 0.05, table 1). In unperturbed walking, the force–length potential was significantly higher and the force–velocity potential was lower compared with the adapted perturbation and hole negotiation (all p < 0.04), without differences in the overall force–length–velocity potential (all p > 0.05, table 1). During the MTU shortening phase, the force–length, force–velocity, and force–length–velocity potentials were reduced (0.43–0.91), with no significant mean differences between the two perturbations and the hole negotiation gait. In unperturbed walking, the force–length potential was higher than in the other three tasks and the force–length–velocity potential was higher than in the adapted perturbation and hole negotiation (all p < 0.003, fig. 8, table 1).

The effect sizes of all variables and all separate task comparisons are shown in supplementary Table 2.

## Discussion

In the present study, we investigated how muscle activation, MTU decoupling mechanisms, and contractile conditions interplay for an effective CoM energy management during unperturbed walking, unpredictable, and adapted drop-like walking perturbations as well as during hole negotiation gait. The peak-to-peak range of the total CoM energy was negligible during unperturbed walking but substantial in the unpredictable and adapted drop-like perturbation as well as hole negotiation during the stance phase of the perturbed leg, indicating energy absorption and production by the musculoskeletal system. In response to the unpredictable perturbation, the soleus muscle EMG activity rapidly increased after the initiation of the plate drop and remained at a high level during the first part of stance, i.e., energy absorption phase. Despite the lengthening of the MTU during this phase, the fascicles exhibited an almost isometric behavior close to L_0_, which confirmed our first hypothesis. This indicates a strut-like muscle behavior and energy conservation within the soleus MTU under optimal conditions for force generation. Experience-based and anticipatory adjustments in the EMG activity were associated with active soleus fascicle lengthening after an initial isometric contraction during the adapted perturbation and hole negotiation gait. The isometric-lengthening behavior indicates the involvement of tendon energy buffering mechanisms and contractile energy dissipation, increasing the effectiveness of CoM energy absorption, confirming also our second hypothesis. Together the findings suggest that a scaled muscle activation regulates muscle stiffness, which tunes the decoupling of muscle fascicles and MTU length changes due to tendon elasticity in the two perturbations and hole negotiation gait task.

During the unpredictable perturbation, the plate was released shortly after level touchdown (~40 ms) during double foot contact. Following touchdown in the hole, the total CoM energy decreased by 13%, indicating energy absorption by the musculoskeletal system. The energy absorption phase was accompanied by substantial lengthening of the soleus MTU (~23 %L_0_). Yet, the soleus muscle fascicles operated with only small length changes (~1.6 %L_0_) on the ascending limb of the force–length curve and close to L_0_ (fig. 4). The nearly isometric behavior of the soleus fascicles during MTU lengthening (fascicle velocity was not significantly different to zero) demonstrates a higher stiffness of the fascicles compared with the tendon, which restricted active fascicle lengthening by decoupling the fascicles from the MTU through tendon elongation. The stiffness of a muscle can be regulated by muscle activation. In fact, we observed an increase in EMG activity starting at 50–80 ms after the unexpected plate drop initiation and a subsequent high EMG activity at touchdown and throughout the MTU lengthening phase (fig. 4). Thus, a fast increase and high muscle activation during MTU lengthening suggestively regulated muscle stiffness and promoted an isometric fascicle behaviour as a result of tendon elasticity, i.e., strut-like muscle behaviour. Because the perturbation was unpredictable and has not been experienced yet, the participants could not rely on any feedforward adjustments, suggesting that the rapid onset in EMG activity was mediated by a reactive response mainly through subcortical neural pathways [79–81]. In addition to the high EMG activity during the MTU lengthening phase (0.73 EMG/EMG_max_), the operating fascicle length close to L_0_ and low fascicle velocity reflect high soleus fascicle force–length and force–velocity potentials (λ_l_ = 0.93 and λ_v_ = 0.96), which facilitated force production and energy conservation within the MTU.

The unpredictable drop of 15 cm was an intense perturbation, in which the musculoskeletal system suddenly absorbed substantial CoM energy in a short time interval of ~ 120 ms, without involvement of experienced-based and anticipatory feedforward control. The rapid reactive increase in EMG activity and the strut-like behaviour of the muscle after the unpredictable drop-like perturbation indicate an interaction between lower-level sensorimotor pathways and the intrinsic properties of the soleus muscle and the Achilles tendon. This interaction may reduce the need for higher-level neural control, thereby simplifying body energy management and reducing sensorimotor delays. Reactive muscle stiffening as a result of fast and high activation presumably improved the management of CoM energy absorption in unpredictable drop-like perturbation scenarios, where anticipatory and predictive adjustments are not possible. On the other hand, the strut-like behavior restricted the absorption of energy by the contractile elements. While this may prevent muscle damage caused by excessive active lengthening [26–28] and promote energy conservation within the MTU, the isometric contraction coupled with the high EMG activity suggests an elevated load of the Achilles tendon. This load could reach detrimental levels upon repeated exposure, increasing the risk of tendon overuse [82]. The increased risk of tendon overloading as a consequence of the simplification of the motor control through a strut-like muscle behavior could indicate a trade-off in the CoM energy management during unpredictable drop-like perturbations.

We observed an active lengthening (velocity significantly different to zero) of the soleus fascicles during the adapted perturbation and hole negotiation gait at 9–21 %stance and 8–24 %stance (fig. 5), respectively, which implies energy dissipation by the contractile elements of the soleus muscle in these two tasks. In the adapted perturbation, experience-based and anticipatory adjustments in the soleus EMG activity patterns before the drop of the plate were found. The EMG activity was higher in the adapted compared with the unpredictable perturbation prior to plate drop but lower at touchdown in the hole. At the beginning of the stance phase, the increase in EMG activity was accompanied by a nearly isometric contraction of the soleus fascicles, despite MTU lengthening, which suggests that an up-scaled muscle activation increased fascicle stiffness (until 9 %stance). Subsequently, the EMG activity decreased and this was associated with active lengthening of the fascicles. We can argue that a down-scaling of muscle activation in this phase reduced the fascicle stiffness and decreased the decoupling between MTU and fascicles, enabling active fascicle lengthening for energy dissipation by the muscle. The initial isometric contraction, despite the lengthening of the muscle–tendon unit, further demonstrates energy storage in the tendon. The subsequent active lengthening of the muscle fascicle indicates a delayed energy dissipation from the muscle contractile elements. Temporal tendon energy buffering has been proposed as a protective mechanism against rapid fascicle lengthening and long operating lengths at the beginning of the contact phase, which can result in muscle damage [29, 30, 83]. The involvement of the tendon buffering function, in combination with active fascicle lengthening and a decrease in EMG activity during the adapted perturbation, may suggest a reduced tissue loading during the energy absorption phase compared with the unpredictable perturbation.

Anticipatory adjustments, such as a lowered EMG activity and increased plantar flexion (shorter MTU length) at touchdown, were also observed during the hole negotiation task. Increased plantar flexion has been previously suggested as a relevant strategy for energy absorption during step-down tasks [84, 85]. The initially low EMG activity during the hole negotiation task observed here suggests that a low level of activation was sufficient to increase the stiffness of the soleus fascicles, leading again to a delayed fascicle elongation and significant decoupling from the MTU during the early stance phase (fig. 5). The high decoupling despite low activation was likely possible because of the low tendon stiffness at the high plantar flexion (i.e., tendon slackness at short MTU length [86]). Following the initial high MTU decoupling, there was a period from 8 to 24 %stance where the soleus fascicles exhibited active lengthening at a low EMG activity relative to the two perturbation tasks (~20 %EMG_max_). Given the simultaneously high force–length-velocity potential of the soleus muscle fascicles of ~ 0.95, it appears that the contractile elements of the soleus muscle, despite the low level of muscle activation, absorbed energy and contributed to the management of CoM energy during this phase.

In the second part of the stance phase during the unpredictable and adapted perturbation as well as hole negotiation, the total CoM energy again increased, reflecting the elevation of the CoM. In all three tasks, the MTU shortened in this phase, which was accompanied by a second peak of EMG activity and continuous fascicle shortening on the upper ascending part of the force–length curve, demonstrating consistent energy production. The fascicle shortening velocity was less than the MTU shortening velocity due to decoupling mainly by tendon recoil, which also facilitated the operating length and velocity and enabled effective contractile conditions for energy production in all three tasks (λ_L_ = 0.81–0.82 and λ_V_ = 0.52–0.60). The timely pattern of soleus fascicle shortening and EMG activity in the energy production phase of unperturbed walking was comparable to the two perturbations and hole negotiation, yet at a smaller scale because no CoM lifting was required (almost constant CoM range). The similar EMG activity in the two perturbations and hole negotiation tasks and comparable timely pattern with respect to unperturbed walking suggest that the energy production phase during the three tasks was close to the regular behavior and thus possibly less demanding for the CoM management than the initial energy absorption phase, where reactive, experienced-based, and anticipatory adjustments in EMG activity were found.

For ethical reasons, the participants were informed that they would experience a perturbation. Though the trial, type, and time point of the perturbation were unknown, the perturbation was not entirely unexpected, and therefore, we used the term unpredictable. Since all parameters at touchdown on the level plate were not different between unperturbed walking and the unpredictable perturbation, we can assume that the perturbation was indeed unpredictable. Furthermore, an activation-dependent shift in L_0_ towards longer length at decreasing activation levels [87, 88] could influence our calculated force–length potential, which was based on a force–length curve at full activation. We recently showed that activation-dependent shifts in L_0_ of the soleus muscle in vivo are moderate [57]. We also developed an approximation model of the activation dependence [57] and using the current measured normalized EMG activity and fascicle length as inputs, it can be estimated that the force–length potential might be 4–8% less during the phase of MTU lengthening across the different tasks and 11–13% less during MTU shortening (fig. 1 in supplementary material), which would not affect our main findings.

In conclusion, the findings suggest that following the unpredictable drop-like perturbation, a reactive increase of soleus muscle activation stiffened the muscle and enabled effective decoupling by tendon elasticity, which resulted in an isometric (i.e., strut-like) muscle behavior and energy conservation within the MTU, facilitating the sudden CoM energy absorption. During the adapted perturbation and hole negotiation gait, an experienced-based and anticipatory scaling of muscle activation presumably regulated the muscle stiffness, resulting in an active fascicle lengthening following the initial isometric contraction. The isometric-lengthening behavior enabled tendon energy buffering mechanisms and contractile energy dissipation, indicating an improved effectiveness of the CoM energy absorption. Together, the results provide novel insight into an activation-dependant muscle stiffness regulation that contributes to the CoM energy absorption management during unpredictable and predictable drop-like walking perturbations. The findings are relevant for the conceptualization of training interventions to improve stability control and for designing exoskeletons, legged robots, and assistive devices capable of dealing with the complex and variable demands of real-world environments.

## Supplementary Information

Below is the link to the electronic supplementary material.

Supplementary file1 (DOCX 262 kb)

## Data Availability

The dataset can be accessed online: 10.6084/m9.figshare.30739739.
