# Supplementary material for: Soleus Muscle Stiffness is Regulated by Scaled Activation to Manage Unpredictable and Predictable Walking Perturbations
Source: Ann Biomed Eng. 2025 Dec 12;54(3):750–66. doi: 10.1007/s10439-025-03928-3 (PMC12960456; doi:10.1007/s10439-025-03928-3)
Supplement: Supplementary file 1 — Supplementary file1 (DOCX 262 kb) [file 10439_2025_3928_MOESM1_ESM.docx]

**Supplementary material_table 1** Definition of measured and calculated parameters.

| **Parameter** | **Definition** |
| --- | --- |
| Stance time (ms) | Contact time of the perturbed right foot with the floor. In the unpredictable and adapted perturbation as well as hole negotiation gait this refers to the contact time in the hole. |
| Plate initiation (ms) | Time interval from touchdown of the perturbed right foot on the level plate to the drop release of the plate. |
| Plate drop time (ms) | Time interval from release of the plate to the touchdown of the perturbed right foot in the hole. |
| MTU lengthening phase (ms or %stance) | Time interval from touchdown of the perturbed right foot (see ‘stance time’) to the maximal length of the muscle-tendon unit during the stance phase. |
| MTU shortening phase (ms or %stance) | Time interval from maximal length of the muscle-tendon to toe-off of the perturbed right foot. |
| CoM total energy (J/kg) | Sum of kinetic and potential energy of the centre of mass. |
| Peak-to-peak range of the CoM energy (J/kg) | Minimum to maximum range of the total centre of mass energy in a predefined time interval. |
| Dorsiflexion angle (°) | Ankle angle in dorsiflexed direction, i.e. negative values. |
| Plantar flexion angle (°) | Ankle angle in plantar flexed direction, i.e. positive values. |
| MTU length (L/L_0_) | Length of the soleus muscle-tendon unit from origin to insertion. The initial length was taken from a regression equation based on cadaver data, considering the individual shank length. Length changes of the muscle-tendon unit were then calculated as product of the instantaneous measured Achilles tendon lever arm and ankle joint angle changes. |
| MTU length changes (L/L_0_) | Minimum to maximum range of the soleus muscle-tendon unit length in a predefined time interval. |
| MTU velocity (V/V_max_) | Soleus muscle-tendon unit velocity. |
| Fascicle length (L/L_0_) | Defined here as the length of an average soleus muscle fascicle between the upper and deeper aponeurosis (fig. 2). |
| Fascicle length changes (L/L_0_) | Minimum to maximum range of the soleus muscle fascicle length in a predefined time interval. |
| Optimal fascicle length (L_0_) | Soleus muscle fascicle length where the force generation of the soleus muscle was greatest according to the experimentally determined fascicle force-length relationship (fig. 2). |
| Fascicle velocity (V/V_max_) | Velocity of the soleus muscle fascicle length. |
| F_max_ (N) | Maximal force of the soleus muscle acting on the Achilles tendon according to the experimentally determined fascicle force-length relationship (corresponding to L_0_). |
| Maximal shortening velocity (V_max_) | Maximal unloaded shortening velocity of the soleus muscle. Estimated based on the measured optimal soleus fascicle length and literature reports of human muscle fiber maximal shortening velocities under physiological temperature conditions. Adjusted for the specific fiber type distribution of the soleus muscle. |
| Pennation angle (°) | Angle of the soleus average muscle fascicle to the deeper aponeuroses (fig. 2). |
| DC_Tendon_ | Decoupling coefficient that quantifies the decoupling of the soleus muscle belly length changes from that of the muscle-tendon unit due to tendon elasticity. |
| DC_Belly_ | Decoupling coefficient that quantifies the decoupling of the soleus muscle fascicle length changes from that of the muscle belly due to fascicle rotation (architectural gear ratio). |
| DC_MTU_ | Decoupling coefficient that quantifies the decoupling of the muscle fascicle length changes from that of the muscle-tendon unit considering both components, i.e. fascicle rotation and tendon elasticity. |
| EMG (EMG/EMG_max_) | Electromyographic activity of the soleus muscle. |
| EMG_max_ | Maximal electromyographic activity of the soleus muscle normalized to the individual maximum EMG activity obtained during a maximum isometric plantar flexion contraction at 0° ankle joint angle. |
| Force-length potential | Fraction of the soleus maximum force according to the force-fascicle length relationship. |
| Force-velocity potential | Fraction of the soleus maximum force according to the force-fascicle velocity relationship. |
| Force-length-velocity potential | Product of the force-length and force-velocity potential. |

**Supplementary material_table 2:** Effect sizes for the separate task comparisons corresponding to table 1 in the main document. Effect size in ‘unpredictable’ refers to comparison with ‘unperturbed’. Effect sizes in ‘adapted’ refer to comparison with ‘unperturbed’ (first value) and ‘unpredictable’ (second value). Effect sizes in ‘hole negotiation’ refers to comparison with ‘unperturbed’ (first value), ‘unpredictable’ (second value) and ‘adapted’ (third value). Effect sizes of 0.2 ≤ d < 0.5 are referred to as small, 0.5 ≤ d < 0.8 as medium, and d ≥ 0.8 as large.

|  | ***MTU lengthening*** | | | | ***MTU shortening*** | | | |
| --- | --- | --- | --- | --- | --- | --- | --- | --- |
|  | **Unperturbed** | **Unpredictable** | **Adapted** | **Hole negotiation** | **Unperturbed** | **Unpredictable** | **Adapted** | **Hole negotiation** |
| Duration (ms) | - | 1.75 | 1.27, 0.48 | 0.36, 1.39, 0.91 | - | 0.57 | 0.53, 0.04 | 0.26, 0.31, 0.27 |
| ∆ L_MTU_ (L/L_0_) | - | 0.06 | 0.55, 0.61 | 3.40, 3.45, 2.84 | - | 1.79 | 1.34, 0.45 | 1.33, 0.47, 0.02 |
| V_MTU_ (V/V_max_) | - | 2.56 | 2.09, 0.47 | 2.54, 0.02, 0.45 | - | 0.41 | 0.91, 0.50 | 1.12, 0.70, 0.21 |
| EMG (EMG/EMG_max_) | - | 2.95 | 1.29, 1.66 | 0.18, 2.77, 1.11 | - | 1.94 | 2.20, 0.26 | 2.16, 0.22, 0.04 |
| ∆ L_fascicle_ (L/L_0_) | - | 1.02 | 1.79, 0.77 | 2.47, 1.44, 0.68 | - | 1.29 | 2.02, 0.73 | 2.32, 1.03, 0.30 |
| V_fascicle_ (V/V_max_) | - | 0.47 | 1.40, 0.93 | 1.45, 0.98, 0.05 | - | 0.24 | 0.62, 0.38 | 0.70, 0.46, 0.08 |
| DC_tendon_ | - | 2.11 | 1.26, 0.85 | 1.57, 0.54, 0.31 | - | 0.96 | 1.30, 0.34 | 1.43, 0.47, 0.13 |
| DC_belly_ | - | 0.39 | 0.70, 0.31 | 1.20, 0.80, 0.49 | - | 0.74 | 0.90, 0.16 | 0.95, 0.21, 0.05 |
| DC_MTU_ | - | 2.21 | 1.31, 0.91 | 1.68, 0.53, 0.37 | - | 0.86 | 1.22, 0.36 | 1.34, 0.48, 0.12 |
| λ_L_ | - | 0.43 | 0.68, 0.25 | 0.85, 0.42, 0.17 | - | 0.81 | 0.91, 0.10 | 0.82, 0.00, 0.10 |
| λ_V_ | - | 0.50 | 1.21, 0.71 | 1.13, 0.63, 0.08 | - | 0.06 | 0.49, 0.42 | 0.64, 0.57, 0.15 |
| λ_LV_ | - | 0.28 | 0.84, 0.56 | 0.66, 0.38, 0.19 | - | 0.44 | 0.92, 0.48 | 0.97, 0.53, 0.05 |

|  | **Unperturbed** | **Unpredictable** | **Adapted** | **Hole negotiation** |
| --- | --- | --- | --- | --- |
| Stance time (ms) | - | 2.99 | 1.87, 1.13 | 0.23, 2.76, 1.64 |


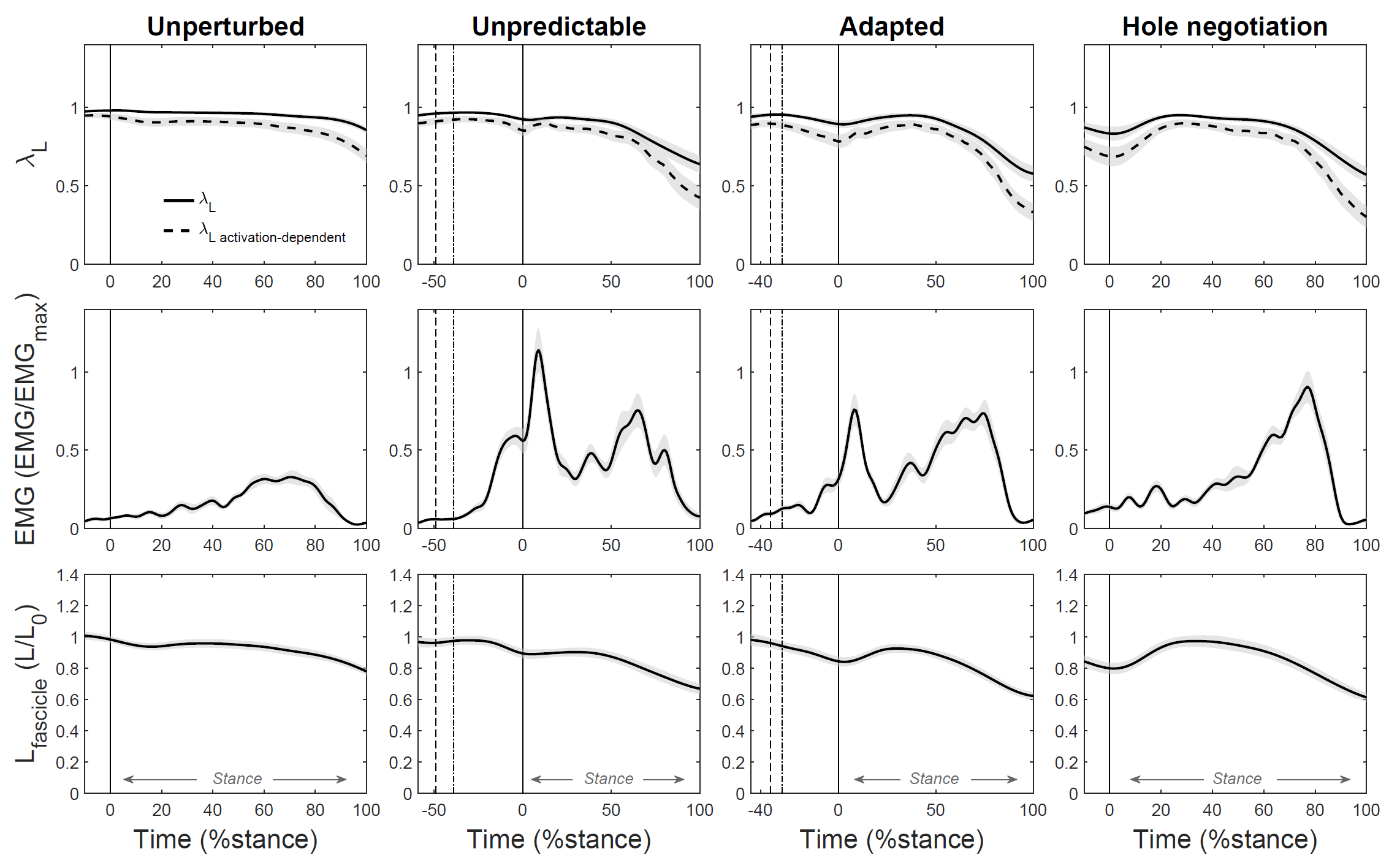


**Supplementary material_Fig. 1** Soleus muscle force-length potential without (λ_L_) and with (λ_L activation-dependent_) consideration of activation-dependent shifts in optimal length of the soleus muscle, EMG activity and soleus muscle fascicle length during unperturbed level walking (column left), the unpredictable drop-like walking perturbation (mid-left column), the adapted walking perturbation (mid-right column) and the hole negotiation (right column). During perturbations (mid panels), the first dotted line indicates the touchdown on the plate and the second dashed line the drop initiation of the plate. The solid vertical line indicates the right touchdown, i.e. after the plate drop in the hole during perturbations. The stance phase was defined from touchdown until toe off (0-100 %stance, n=19, mean±standard error).

**Supplementary figure belongs to:**

Soleus muscle stiffness is regulated by scaled activation to manage unpredictable and predictable gait perturbations

Sebastian Bohm^1,2^, Morteza Ghasemi^1,2^, Christos Theodorakis^1,2^, Falk Mersmann^1,2^, Thomas Roberts^3^, Adamantios Arampatzis^1,2^

^1^ Humboldt-Universität zu Berlin, Department of Training and Movement Sciences, Berlin, Germany

^2^ Berlin School of Movement Science, Humboldt-Universität zu Berlin, Berlin, Germany

^3^ Brown University, Division of Biology and Medicine, Providence, US

Corresponding author: Sebastian Bohm, Email: sebastian.bohm@hu-berlin.de

Annals of Biomedical Engineering
